# Methodic aspects of influenza and respiratory syncytial virus detection in raw wastewater and presence in treatment plants in southeastern Germany

**DOI:** 10.1038/s41598-025-13998-x

**Published:** 2025-08-02

**Authors:** Michael Geissler, Helene Berndt, Ella Herberger, Konrad Wilms, Roger Dumke

**Affiliations:** Institute of Medical Microbiology and Virology, University Hospital Carl Gustav Carus, Technische Universität Dresden, Dresden, Germany

**Keywords:** Wastewater, Monitoring, Virus detection, Wastewater-based epidemiology, Influenza virus, Respiratory syncytial virus, Environmental microbiology, Virology

## Abstract

During the COVID-19 pandemic, wastewater-based epidemiology (WBE) has been developed as an additional tool to follow epidemiological trends in the catchment area of treatment plants. Meanwhile, further viral agents of respiratory infections were included in monitoring programs. However, differences in sample processing may impair the results comparison among studies. With identical virus strains, we investigated different concentration methods, RNA isolation kits and primer/probe combinations for detection of influenza virus (IV) A/B and respiratory syncytial virus (RSV) A/B. For procedure optimization, virus enrichment and RNA extraction methods were found to be of relevance. Conversely, the detection step was identified to have a relatively low influence. Using standardized protocols, 24 h composite samples from eight wastewater treatment plants (WWTP) in southeast Germany (2x/week) were analyzed (January 2024 to December 2024) for IVA/B and RSV-A/B. The following rates of virus-positive samples were determined: 20.5% (IVA including 2.2% H1N1pdm09), 4.5% (IVB), 32.6% (RSV-A) and 2.4% (RSV-B). IV and RSV detections showed a matching trend when compared to reported cases. This study contributes to a better understanding of factors influencing the detection of IV and RSV in wastewater as well as in the epidemiological significance of virus monitoring in WWTPs.

**Supplementary Information:**

The online version contains supplementary material available at 10.1038/s41598-025-13998-x.

Stool-excreted bacteria, parasites and viruses are introduced into wastewater by infected individuals. Thus, wastewater monitoring can provide valuable insights on pathogen circulation in the population covered by the wastewater treatment plant (WWTP). In contrast to the resource-intensive clinical surveillance, results of wastewater-based epidemiology (WBE) do not depend on the number of reported cases in the population. Additionally, clinical testing often misses asymptomatic infections which contribute to the continuous spread of the pathogen. Wastewater testing is not affected by the infection symptomatology, making it an important complementary tool to clinical monitoring. For decades, monitoring of waters for infectious agents was limited to classical enteric pathogens including bacteria, like *Salmonella* spec. and *Vibrio cholera*, and enteric viruses (e.g., norovirus, rotavirus, poliovirus)^1,2^. In 2011, a study was published^3^ firstly confirming the presence of a primarily respiratory virus (influenza virus A (IVA)) in sewage influents, effluents and river water. But only during the COVID-19 pandemic, WBE has been developed as valuable additional instrument to follow the epidemiological situation of a respiratory infection in the population^4^. Meanwhile, the stool excretion of other respiratory viruses was investigated^5,6^. In addition, the spectrum of target organisms in WBE approaches can be expanded to cover different aspects of local and regional public health. Ahmed et al.^7^ described the detection of more than 12 different respiratory viruses and confirmed the occurrence of most of these viruses in Australian wastewaters. Kim et al.^8^ combined eight respiratory viruses with pneumonia-causing bacteria and enteric viruses in a great panel of pathogens in a wastewater surveillance system in South Korea. Despite the challenges in establishing associations between wastewater and clinical data and the confirmation of a stable anticipation time of virus detection in wastewater for presenting an early warning tool, these studies confirm the potential of wastewater monitoring to follow the epidemiology of diverse agents of infectious diseases. Especially, IVA/B and respiratory syncytial viruses (RSV) have been developed time-dependent peaks of incidence including the occurrence of severe courses of infections in patients both regionally and globally. Summarizing the available data, these viruses are described as stool-excreted by 36% (IV) and 14% (RSV) of infected people^5^. In a recent study, Zhang et al.^6^ reported an excretion rate of 40.5% in patients with confirmed RSV infection. The presence of both viruses in sewage influents have been confirmed in many countries^9–14^. However, results of studies are inconsistent probably not only due to local differences in the circulation of viruses but also to the use of many different methods to concentrate and detect IV and RSV. Even though these viruses have been found in high concentrations in sludge and wastewater solids^15–17^, it is preferred in many studies to first remove the solids from wastewater before analysis. For virus concentration from wastewaters, procedures based on precipitation^10,18^, adsorption/elution^7,11,19^, ultrafiltration with different filter systems^20–22^, and commercial systems for enrichment^8,23,24^ have been applied. Similarly, a wide range of methods are used to extract RNA from the concentrated samples. Based on the results with other virus species^25^, differences in the recovery rates depending on the concentration and extraction methods used are expected. However, corresponding reports for IV and RSV detection in wastewater samples are rare.

In the present study, we compared procedures for concentration of IVA, IVB, RSV-A and RSV-B, various kits for RNA extraction and primer/probe combinations to optimize the detection of these viruses in wastewater samples. Using a standardized and validated method, presence of viruses in eight WWTPs in Germany was tested during a one-year monitoring period. This approach allows an evaluation of current methods as well as a post-pandemic comparison of the circulation of two important respiratory viruses in an urban region.

## Results

### Comparison of methods for detection of IVA/B and RSV-A/B

**Fig. 1:**
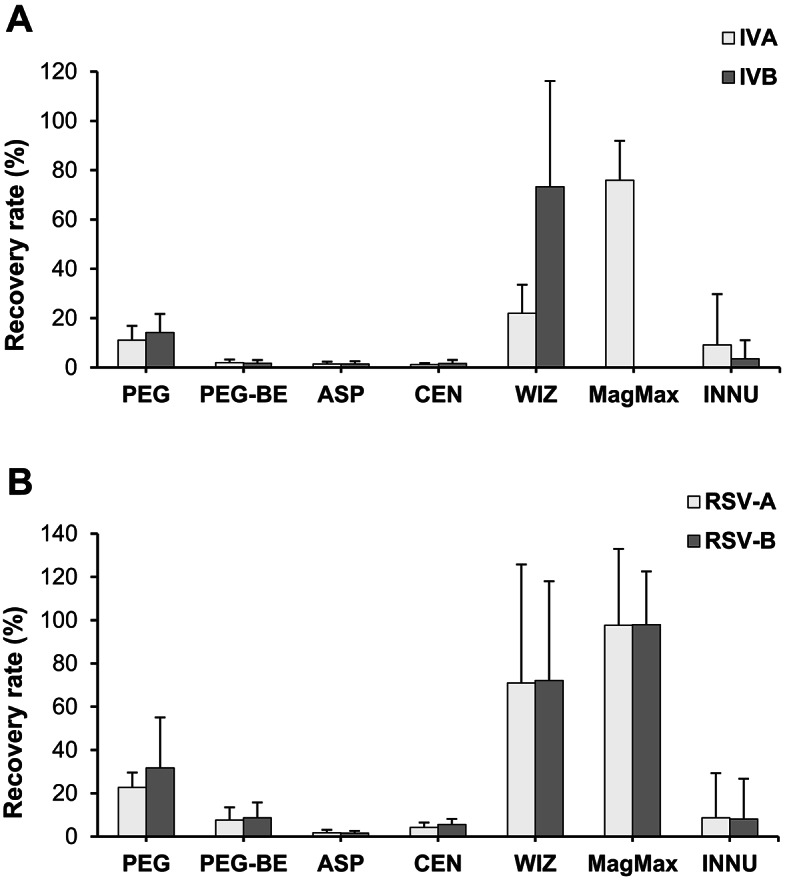
Recovery rates of spiked IV (**A**) and RSV (**B**) in pooled raw wastewater samples after concentration with different methods, Mean and standard deviation of eight independent experiments, PEG: polyethylene glycol 8000 precipitation, PEG-BE: polyethylene glycol 8000 precipitation with beef extract elution, ASP: ammonium sulfate precipitation, CEN: ultrafiltration with Centricon columns, WIZ: concentration with the Wizard Enviro TNA kit (Promega), MagMax: concentration with the MagMax Wastewater Ultra kit (Applied Biosystems; due to technical problems, data for the concentration of IVB are not available), INNU: concentration with the innuPrep kit (Innuscreen).

The recovery rates of the seven procedures to concentrate IVA/B and RSV-A/B from raw wastewater samples are summarized in Fig. 1. The following virus concentrations were measured in the spiked wastewater samples before enrichment: 7.8 × 10^6^/ml-5.4 × 10^7^/ml (IVA), 1.5 × 10^6^/ml-3.1 × 10^7^/ml (IVB), 2.1 × 10^6^/ml-2.3 × 10^7^/ml (RSV-A) and 2.8 × 10^6^/ml-6.5 × 10^7^/ml (RSV-B), respectively. Overall mean recovery rates ranged between 1% (ASP/CEN, IVA and B) and 98% (MagMax, RSV-A and B). In general, only slight differences (except for the WIZ and INNU kit and IV) between IVA and B as well as between RSV-A and B were measured indicating a low impact of specific properties of the genotypes of the two virus species on the efficiency of enrichment procedures. The same is the case for the rough order of mean efficiencies: CEN/ASP/PEG-BE (only small differences of rates between each other; recovery < 9%) < INNU/PEG (recovery: 8–23%) < WIZ/MagMax (recovery: 22–98%). It is important to note that for different methods (e.g., WIZ, MagMax, INNU) relatively great standard deviations were determined reflecting a strong inter-experimental variation. PEG 8000 precipitation which is used for the surveillance of viruses in the WWTPs, resulted in mean recoveries of 11% (IVA), 14% (IVB), 23% (RSV-A) and 32% (RSV-B), respectively. Except for PEG and INNU (IVA and RSV-A), differences between the recoveries after PEG precipitation and the other concentration methods are statistically significant (t-test, *p* < 0.05; Table S1).

**Fig. 2:**
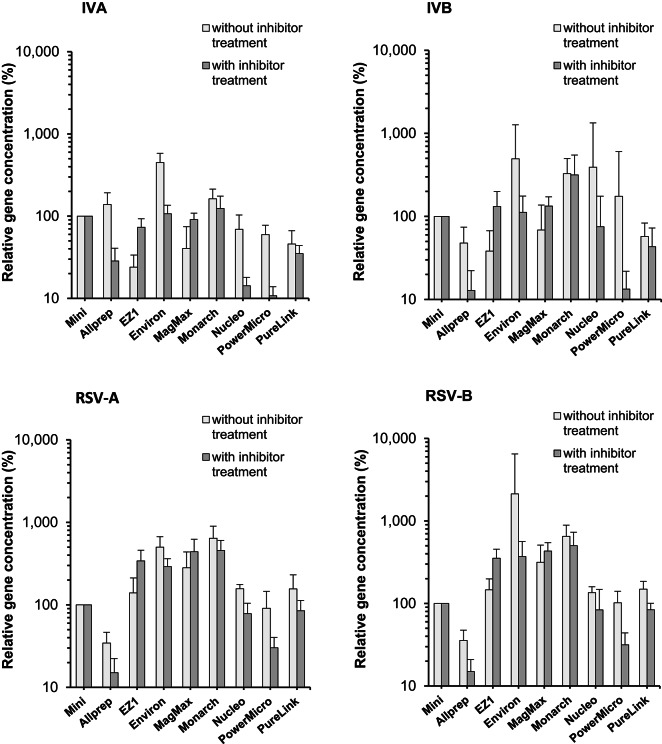
Relative efficiency (results of the RNeasy kit was set 100%) of different methods to extract RNA from PEG-concentrated samples spiked with IVA/B and RSV-A/B. Mean and standard deviation of eight independent experiments. Mini: RNeasy Mini kit (Qiagen); AllPrep: AllPrep Power Viral DNA/RNA kit (Qiagen); EZ1: EZ1 Virus Mini kit v2.0 (Qiagen); Environ: Environ Water RNA kit (Zymo); MagMax: MagMax Wastewater Ultra kit (Applied Biosystems); Monarch: Monarch Total RNA Miniprep kit (New England Biolabs); Nucleo: Nucleo Spin kit (Macherey & Nagel); PowerMicro: RNeasy PowerMicrobiome kit (Qiagen); PureLink: PureLink RNA Mini kit (Invitrogen).

Regarding the reference method of RNA isolation used for the monitoring of viruses (RNeasy, set to 100%), the results of the performance of the other eight procedures for RNA isolation can be found in Fig. 2. The following ranges of genome concentrations were measured in the different RNA preparations: 6.8 × 10^2^/ml − 1.1 × 10^6^/ml (IVA), 6.6 × 10^0^/ml − 5.3 × 10^5^/ml (IVB), 9.7 × 10^2^/ml − 2.1 × 10^6^/ml (RSV-A) and 2.3 × 10^1^/ml − 4.0 × 10^5^/ml (RSV-B), respectively. Despite genotype-specific differences in some methods (e.g., Nucleo and IV), use of most procedures resulted in a comparable picture of relative gene concentrations between IVA and B as well as between RSV-A and B. Interestingly, relevant differences in the results were observed after comparison of samples with and without performing a step to remove PCR inhibitors. Without inhibitor treatment, consistently higher efficiencies related to RNeasy were found for the Environ and the Monarch kit whereas other procedures were confirmed as more effective for some/one of the virus types only (e.g., MagMax and RSV). Of note, the automatically processed EZ1 kit showed a relatively low efficiency, particularly for IVA and IVB. Comparison of data after inhibitor treatment confirmed a better performance of RNeasy and EZ1 related to other methods indicating a stronger sensitivity of both kits to the influence of PCR inhibitors. To a lesser extent, this association could be found for RSV-A/B. For distinct kits (e.g., Allprep, PowerMicro), efficiency remained low regarding all four virus types tested. The heterogeneity of variance hampers the use of t- and ANOVA-tests to calculate statistical significances. Without removal of PCR inhibitors, the following factors between the methods with the highest and lowest efficiency were calculated: 18.7 (IVA, EZ1 vs. Environ), 13.0 (IVB, EZ1 vs. Monarch), 59.1 (RSV-A, Allprep vs. Monarch) and 18.1 (RSV-B, Allprep vs. Environ). With the treatment step to reduce inhibitory substances, factors ranged between 11.3 (IVA, PowerMicro vs. Monarch) and 33.4 (RSV-B, Allprep vs. Monarch).

**Fig. 3:**
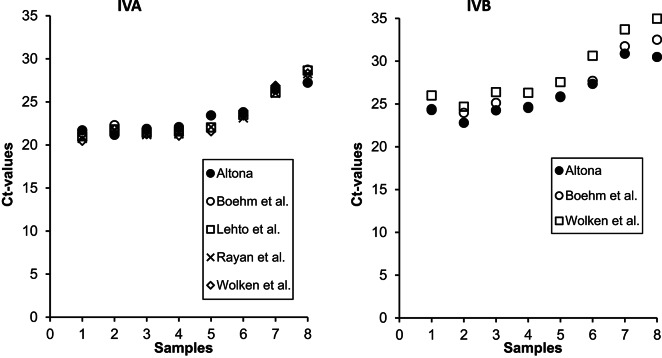
Results of different primer/probe combinations for detection of IVA and B. Mean of two replicates per sample.

Results of testing different primer/probe combinations for detection of IVA and IVB can be found in Fig. 3. After using five combinations for detection of IVA in pooled RNA preparations from spiking experiments, Ct values slightly differed. In comparison with the reference method RealStar Influenza Screen & Type RT-PCR kit, the following mean quotients of Ct values between the reference method and the different primer/probe combinations were found (*n* = 8): 1.002 ± 0.038^15^, 0.990 ± 0.035^22^, 0.983 ± 0.042^26^ and 0.982 ± 0.034^27^. For detection of IVB, nearly the same differences were obtained for the comparison of both published methods with the commercial kit: 1.092 ± 0.028^26^ and 1.026 ± 0.024^15^.

### Monitoring of IVA/B and RSV-A/B

**Fig. 4:**
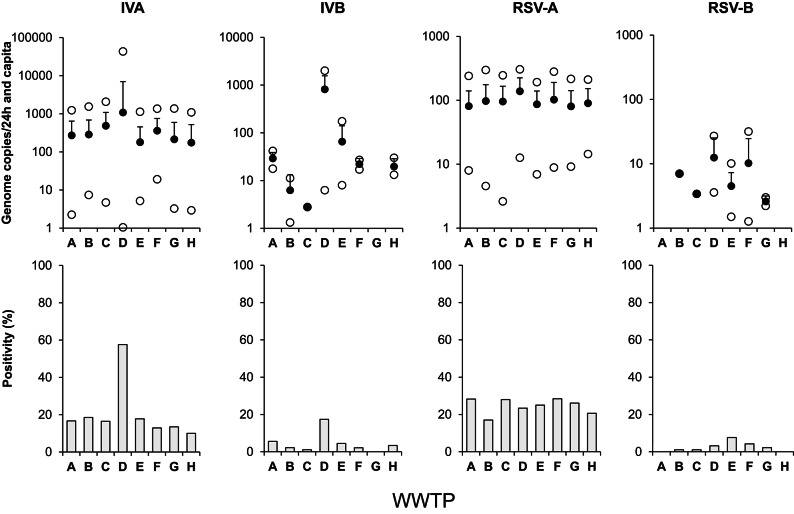
Mean (filled circles), maximum and minimum (open circles) of normalized concentrations of virus copies of virus-positive samples (**A**) and positivity rate (**B**) of different viruses in eight wastewater treatment plants (WWTP) in southeast Germany, January to December 2024.

Between January and December 2024, eight WWTPs in southeastern Germany were included in the surveillance program of this study (Fig. 4, Fig. S1-S4). In 20.5% of samples (*n* = 727), the presence of IVA RNA was confirmed (mean: 381.2 gc/dxc; range: 1.0–43,185.3 gc/dxc). In contrast, RNA of IVB was found in 4.5% of samples (mean: 137.3 gc/dxc; range: 1.3–1.999,9 gc/dxc) and only in WWTPs A-B, D-F and H. Regarding IVA/B, a differing pattern of virus presence was found in wastewater of plant D showing relatively high numbers of genome copies throughout the year. RSV-A could be detected in 32.6% of samples (mean: 96.4/dxc; range: 2.6–304.7/dxc) whereas the rate of RSV-B-positive samples was lower (2.4%; mean: 6.6 gc/dxc; range: 1.3–31.7 gc/dxc). In most cases, positive samples were found in the first half of the year in six of the eight plants. Mainly in December 2024, the presence of RNA of H1N1pdm09 was confirmed with low frequency (2.2%; 5–207 gc/dxc) in six of the eight WWTPs (data not shown).

**Fig. 5:**
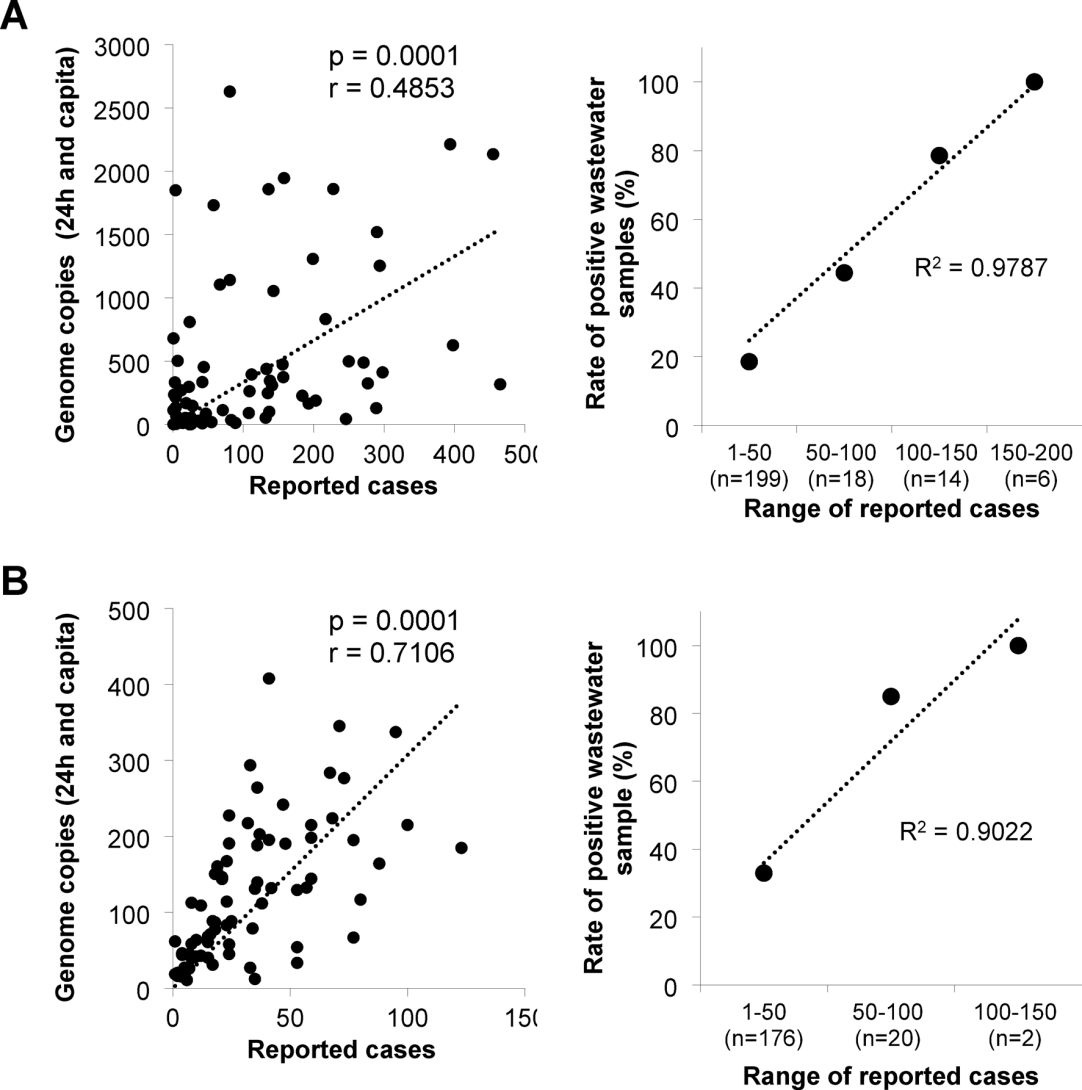
Association of viral genome copies in wastewater with reported cases in different catchments of WWTPs A-H, January to December 2024. A - IV, B - RSV. p: p-value, r: coefficient of Spearman’s rank correlation.

Association of number of genome copies detected in wastewater with cases of influenza and RSV infections in the catchment of different WWTPs is shown in Fig. 5. From the beginning of 2024, laboratory-confirmed cases of IV and RSV steadily raised until week 6 and then dropped until week 20. Especially in catchments of WWTPs D and E/G, cases of IV and, to a lesser extent, RSV infections were rising at the end of the wastewater sampling campaign (weeks 48 to 51; Fig. S5). During April to November 2024, determination of IVA/B and RSV-A/B remained below the LOD in most WWTPs. However, overall correlation between the normalized number of genome copies in wastewater and reported cases in the corresponding catchment areas of all WWTPs is statistically significant (Fig. 5A, table S1). In Fig. S6 and S7, temporal sections (weeks 3 to 20) are presented confirming a fundamental relationship of numbers of IV and RSV infections and wastewater data in this period. Classification of numbers of reported cases in clusters and their relation to the rate of virus-positive detection in the timely corresponding wastewaters can be found in Fig. 5B. The rate of IV- and RSV-positive water samples linearly increased depending on the increase of number of infections in the clusters. However, the association of the number of cases with consistently detection in wastewater was different between both viruses showing a positivity of 100% for 150–200 cases (IV) and for 100–150 cases (RSV) in the catchment. In contrast, the rate of positive virus detection was 19% (IV) and 33% (RSV) if only 1–50 infections were reported. Using a detailed view on the period of increasing cases of reported influenza infections in November/December 2024 (Fig. S8), mean viral genome copies in wastewater of WWTPs B + H and E + G increased accordingly. It is important to note that, except for catchment of WWTPs E + G, the number of reported cases remained below 30 during this period. According to our data, it is difficult to define a lead time of wastewater monitoring data for the prediction of rising numbers in clinically confirmed influenza infections. Despite a slight increase of reported RSV cases in the catchment of some WWTPs (< 40 confirmed infections), the detection of specific genome copies remained negative in most wastewater samples collected during this period.

## Discussion

In this study, we combined the monitoring of IVA/B and RSV-A/B in eight WWTPs with a comparative study of methods to process samples and to detect these viruses in wastewater. Our goal was an evaluation of the monitoring procedure by direct comparison with other approaches using sewage samples from the same plants. All methods of virus enrichment and RNA preparation which were introduced in the present report are already tested, used in or intended for virological investigations of wastewater. The data confirmed that great differences occur regarding the methods for concentration of viruses and for RNA preparation but not to that extent for the primer/probe combination. This result is important for further efforts to standardize the methods established in virus monitoring programs based on WBE and for comparison of results of studies. Regarding the enrichment procedures, the data for the four viruses showed a tendency to higher recovery rates of commercial kits (WIZ, MagMax) followed by PEG precipitation (Fig. 1). In comparison to the results of other studies, it can be assumed that the chemical and biological quality of wastewater used for the experiments might have a strong influence on the recovery which is experimentally confirmed for many viruses for decades. Most of the tested methods are based on adsorption of viruses to different materials. The process can be influenced by the variable composition of the wastewater matrix including the role of non-influenceable factors, like meteorological events and time-dependent discharge of industrial wastewater. This makes it extremely difficult to clearly determine the connection between sample quality and the efficiency of concentration methods. Using different enrichment methods, Farkas et al.^28^ demonstrated a greater recovery of spiked viruses in deionized water in comparison to wastewater, an improved recovery with reduced sample volume (lower amount of substances in water interfering with the enrichment process) and confirmed the influence of chemical parameters of the sample (e.g., ammonium) on the recovery after concentration with ultrafiltration and PEG-BE. The authors of the study^28^ spiked IVA and B in wastewater and found mean recovery rates < 10% for a comparable volume of processed wastewater (37.5 ml) and a superiority of BE-PEG over PEG, ASP and the InnovaPrep system based on hollow fiber filter tips. Using SARS-CoV-2 and MS2 phage, Gouthro et al.^29^ demonstrated the high recovery of the WIZ kit for processing passive samples in comparison with two other commercial kits. Regarding naturally occurring numbers of genome copies, the experiments in our study were carried out with relatively high concentrations of viruses before enrichment. As confirmed for SARS-CoV-2, IV and RSV^30,31^, this might have an influence on the recovery rate but should not strongly affect the results among the different tested methods. Regarding the RNA isolation, a consistent superiority of the bead-based extraction method (MagMax) over column-based kits could not be confirmed. However, the differences between the tested columns are striking and large enough to consider nucleic acid isolation as important for the efficiency of the whole analytical process. Furthermore, the sensitivity of some kits to the PCR inhibitor treatment is interesting. The negative effect that these substances have on real-time quantitative PCR (RT-qPCR) results is well-known but not fully understood^32^. Further investigations have to clear if a method-specific loss of RNA during sample processing might be a reason for our findings. In contrast to sample concentration and RNA isolation, the detection system was less relevant for IV detection in sewage. The importance of the primer/probe combination for the analytical performance of RT-qPCR or ddPCR in WBE was investigated for SARS-CoV-2^33,34^. To our knowledge, a study comparing different primer and probes for IVA/B detection in sewage was not carried out to date.

Despite the determination of procedures with higher efficiency to concentrate IV and RSV, PEG precipitation was performed for the monitoring of the WWTPs, mainly to be able to largely compare the results with a previous study^35^. PEG precipitation was demonstrated effective not only for the concentration of SARS-CoV-2^31^ but is also used for the enrichment of IV and RSV from wastewater^13,27,36–42^ and was found superior to the novel Nanotrap Microbiome Particles concentration method^43^. Even though we could confirm the presence of IVA/B and RSV-A/B in wastewater of different WWTPs using PEG precipitation, experimental determination of the efficiency of the used method is important for the evaluation of the results obtained. The spiking experiments showed that the recovery of the PEG method for concentration of IVA (11%), IVB (14%), RSV-A (23%) and RSV-B (32%) is variable among the four viruses and lower in comparison with SARS-CoV-2 (59%^44^; underlining the importance of the virus species for the yield rate of an enrichment process^28^. The differing concentration efficiency likely contributes to the higher LOD of IVA/B and RSV-A/B (Table S2) as well as to its quantification when compared to SARS-CoV-2 (LOD: 1,289 copies/l^45^, and will influence frequency of detection and number of genome copies of IV and RSV measured in the wastewater. Despite these expectable differences between viruses, it is foreseeable that WBE will develop its full potential by investigation of different stool-excreted viruses with epidemiological relevance, especially if multiplex procedures are used^30^. For the effectiveness of future monitoring programs it is inevitable that this detection is performed in wastewater samples concentrated by a single procedure. Use of different methods to enrich viruses in wastewater would negatively impact laboratories in terms of both time and cost. Aspects such as performance or processing time are of great importance when deciding which methodology is to be applied. PEG precipitation is easy to handle, inexpensive and allows the parallel processing of samples. On the other hand, the procedure is relatively time-consuming and needs an expensive high-speed centrifuge. For successful detection of numerous pathogens, the suitability of samples processed with only one method has been confirmed^7,8^. However, it is currently not clear which concentration and RNA extraction combination offers the best compromise to analyze a panel of viruses in wastewaters.

Due to the use of the same methods for IV concentration, RNA preparation and detection, the results of the present study can largely be compared with a previous report which included WWTPs C and D (referred to there as 1 and 2^35^,. Whereas in 2022 IVB was detected in 36% and 58% of samples of WWTPs C and D, in 2024 it was only found in 1% and 17% of samples. Furthermore, each WWTP shows a different pattern with punctual detections of IBV during 2024. This is in agreement with different studies reporting strongly variable and time-depending concentrations of IVB RNA in wastewater^8,9,24,46^. Based on the seasonal and regional circulation of influenza viruses, a different presence of both genotypes can be expected in sewage samples. Although less data is available, the same seems to be the case for both RSV genotypes^47^. As reported by Lehto et al.^22^, rate of IV-positive samples increases with the incidence of infections and the cluster of 1–5 IVA cases/100.000 inhabitants/week resulted in around 79% IVA-positive wastewater samples. In our catchments, the range of 1–50 reported influenza cases (19% positivity in sewage) corresponds to mean incidences between 1.4 (WWTPs E + G; range: 0.2–5.6) and 4.4 (WWTPs C and B + H, range: 0.4–19.4) cases/100,000 inhabitants/week. Interestingly, a positive detection of IV and RSV in wastewater corresponds to a mean minimal number of reported cases of 33 (range in the different catchments: 14–50) and 5 (1–13), respectively, indicating a difference between both viruses. Further studies must clarify whether factors like a higher excretion of viruses in RSV- vs. IV-infected patients^6^, differences of the testing/reporting regime of both infectious diseases and/or a higher stability of RSV in comparison with IV in sewage^48^ could be reasons for this finding. However, results of preliminary experiments (14 °C) in wastewater of all eight WWTPs of this report demonstrated mean T_90_ values (time for a 90% reduction) for RNA of IVA/B (26.0 days) and RSV-A/B (19.9 days, data not shown).

Besides other advantages, wastewater monitoring is cost-effective and could be an early warning system in comparison with the data of the clinical surveillance of influenza and RSV infections in the corresponding catchments of WWTPs. In the present study, the confirmation of a lead time of increasing virus concentrations in wastewater in comparison with clinical data was difficult to determine for both viruses and varies between WWTPs and species. Unfortunately, relatively high numbers of reported influenza and RSV cases were already present at the beginning of the monitoring and the next increase of infections was just starting when the wastewater sampling campaign ended. Confirmation of an early warning capacity is important to justify the complementary use of wastewater monitoring with clinical surveillance, this capacity was calculated to around ten days in some studies^13,49^. In contrast, this lead time was not confirmed by other reports, was not consistent between WWTPs or could not be demonstrated for all significant increases of cases^7,10,14,18,22,37,39,40,42,50^. Faherty et al.^23^ found that measured viral genome copies in sewage lagged the results of traditional influenza surveillance by around one week and recommend a wastewater monitoring for catchments areas with limited clinical data. Differences in the frequency of sewage sampling, in the regional testing/reporting system and in the immunization rate of the population might be reasons for varying observations.

In conclusion, the results of the present study provide experimental data for an optimization of methods used in monitoring programs to detect two important respiratory viruses in wastewater. However, the results of the performed surveillance in eight WWTPs confirmed the successful detection of IV and RSV by using methods with an averaged but pre-tested efficiency.

## Methods

### Preparation of viruses for the spiking experiments

Virus strains (IVA: H1N1pdm09; IVB: Victoria; RSV-A: ON1.1 18-3723; RSV-B: BA9-I 18–0564) were obtained from the Robert Koch-Institut, Berlin, Germany, and were propagated in HEp2 (MEM with GlutaMAX and 10% fetal bovine serum) and A549 cells (ATCC CCL-185, DMEM with GlutaMAX and 10% fetal bovine serum) according to standard procedures. Supernatants in cell culture flasks with clearly visible cytopathic effect were aliquoted after careful mixing, inactivated (60 °C, 30 min) and storaged (−80 °C). Before use in spiking experiments, RNA of an aliquot was extracted with the RNeasy Mini kit (Qiagen, Hilden, Germany) and the number of genome copies was determined as described below. Virus propagation, handling of virus stocks and of spiked wastewater samples were strictly separated from processing of monitoring samples.

### Wastewater treatment plants and sampling

Surveillance of IVA/B and RSV-A/B was performed in eight WWTPs of different size located in the federal state Saxony, southeast Germany. Main characteristics of plants are summarized in Table 1. Tested WWTPs cover around 43% of the population of Saxony. In the period from January to December 2024, 24 h-composite samples were taken twice per week (Monday and Wednesday) in all WWTPs and transported refrigerated to the laboratory for its immediate processing.

**Table 1:** Characterization of wastewater treatment plants (WWTP).

WWTP	Population served	Mean volume (m³/d)	Combined sewer system	Sewer network length (km)	Ratio industrial: municipal wastewater
A	43,100	7,700	Yes	300	1:19.0
B	11,400	4,200	No	233	1:2.4
C	249,000	77,100	No	597	1:5.7
D	700,000	131,000	No	904	1:1.9
E	27,400	5,400	Yes	50	1:19.0
F	63,900	18,100	Yes	201	1:4.0
G	626,000	91,200	Yes	892	1:3.2
H	40,200	14,500	No	370	1:1.1

### Preparation of wastewater samples

To compare the concentration efficiency of the different methods for IVA/B and RSV-A/B, wastewater samples of all WWTPs were pooled, carefully mixed and divided into aliquots (50 ml). Samples were centrifuged (3,300 g, 25 min, 4 °C; except aliquots for method (b)), spiked with the four virus types and incubated at room temperature with agitation (30 min). From all samples, an aliquot was taken to determine the initial virus concentration after preparation of the RNA using the RNeasy kit. The following enrichment procedures were tested: (a) polyethylene glycol (PEG) precipitation as described^35^. Briefly, after addition of NaCl and PEG 8000, suspension was mixed overhead (room temperature, 25 min) and ultracentrifuged (12,000 g, 1.5 h, 4 °C). Pellets were suspended in phosphate buffered saline (PBS, pH 7.4) resulting in volumes of concentrates between 400 and 600 µl. (b) beef extract modified PEG precipitation (PEG-BE^28^, to elute viruses from solid matter. Samples (50 ml) were mixed with beef extract (3%), adjusted to pH 5.5 and incubated at room temperature (30 min, 60 rpm). Subsequently, the PEG protocol described above ((a), including centrifugation) was followed. (c) ammonium sulfate precipitation (ASP). After centrifugation, ammonium sulfate (40%) was added to the supernatant, the solution was mixed, incubated (4 °C, 1 h) and centrifuged (12,000 g, 4 °C, 90 min). The obtained pellets were dissolved in PBS. (d) ultrafiltration (CEN) with Centricon Plus-70 filters (Merck Millipore, Tullagreen, Ireland) after a modified pre-centrifugation (10,000 g, 4 °C, 10 min). (e) concentration (WIZ) with the Wizard Enviro TNA kit (Promega, Madison, WI, USA). (f) concentration (MagMax) with the MagMax Wastewater Ultra kit (Applied Biosystems, Woodward St. Austin, TX, USA). (g) concentration (INNU) using the innuPrep kit (Innuscreen, Berlin, Germany). Virus enrichment with ultrafilters and procedures (d) to (g) was done according to recommendations of the manufacturer. To evaluate the recovery rate of the different methods, three replicates of RT-qPCR were used to determine the concentration of viral genome copies before and after the enrichment.

Monitoring samples were concentrated by PEG precipitation as described above (method (a)) using the supernatants (45 ml) after centrifugation of 50 ml samples.

### RNA extraction, virus detection and quantification

To investigate their efficacy, different methods to extract the RNA of IVA/B and RSV-A/B were tested. PEG-precipitated samples from the eight monitored WWTPs (confirmed virus-negative after pre-testing) were pooled to create a single representative matrix, which was then spiked and aliquoted. The following RNA isolation assays were compared: (a) RNeasy Mini kit; (b) AllPrep Power Viral DNA/RNA kit (Qiagen); (c) EZ1 Virus Mini kit v2.0 (Qiagen); (d) Environ Water RNA kit (Zymo Research, Irvine, CA, USA); (e) MagMax Wastewater Ultra kit (Applied Biosystems); (f) Monarch Total RNA Miniprep kit (New England Biolabs, Ipswich, MA, USA); (g) Nucleo Spin kit (Macherey & Nagel, Düren, Germany); (h) RNeasy PowerMicrobiome kit (Qiagen; adding IRS for inhibitor removal was included); (i) PureLink RNA Mini kit (Invitrogen, Carlsbad, CA, USA). Except EZ1, methods were manually performed and all were used as recommended by the manufacturers. For most of assays, an elution volume of 50 µl was adjusted (EZ1: 60 µl). To investigate the influence of PCR inhibitors on RNA extraction results, all preparations were divided into two equal aliquots. RT-qPCR was performed on untreated and on inhibitor removal-treated samples (Zymo). Viral genome copies were measured as described below (three replicates per sample). Using a relative comparison (results of the RNeasy kit as reference method were set to 100%), efficiency of procedures was determined.

Regarding the monitoring samples, RNA from 200 µl PEG-concentrate was prepared using RNeasy columns (elution volume: 50 µl) and treated to remove PCR inhibitors as described above.

To detect IVA and IVB, RT-qPCR was performed as reported^35^. Briefly, a commercial kit (Altona Diagnostics, Hamburg, Germany) amplifying the matrix protein gene of IVA and IVB (RealStar Influenza Screen & Type RT-PCR kit 4.0) was used as recommended by the manufacturer. Besides the differentiation of IVA (reporter: FAM) and IVB (Cy5) in the sample, the assay allows a separate detection of H1N1pdm09 (ROX). For testing of RSV-A and B, the RSV RT-PCR Kit 3.0 (Altona Diagnostics) was applied. All amplifications were done in duplicate in a QuantStudio5 thermocycler. Positive, negative and extraction controls (provided with the kits) were included in any run. Samples were considered positive if amplification was positive in both replicates with Ct values ≤ 40. Virus concentrations were calculated using standard curves of commercial RNA standards of IVA and IVB as described^35^ and of the RSV verification kit (LGC Seracare, Milford, MA, USA; Fig. S9). Limit of detection (LOD) of IV and RSV detection was calculated as reported^45^. Briefly, RNA of standards diluted in virus-negative wastewater (pooled samples of WWTPs A-H) was PEG enriched, concentrates were extracted with the RNeasy kit and 95% positivity was determined (10 replicates per dilution).

### Investigation of different primer/probe combinations for the detection of IVA/B

Due to the limited number of different primer and probes described for the detection of RSV-A and RSV-B, we only tested the influence of the detection system on IV. In detail, the primer and probes described in Boehm et al.^15^; IVA/B; corresponding to the CDC recommendations), Lehto et al.^22^; IVA), Raya et al.^27^; IVA) and Wolken et al.^26^; IVA/B) were used and compared with the results of the RealStar Influenza Screen & Type RT-PCR kit. Sequences of primers and probes are summarized in table S3. Pooled samples from spiking experiments after extraction of RNA with the RNeasy kit and removal of PCR inhibitors served as matrices. All RT-qPCR assays (except the RealStar Influenza Screen & Type RT-PCR) contain the following components (25 µl): 6.25 µl TaqMan Fast Virus 1-Step MasterMix (Thermo Fisher), 1 µl primer each (5 pmol), 1 µl probe (2 pmol), 10.75 µl pcr-grade water and 5 µl template, positive control or water and were run in duplicate at the same conditions: 50 °C, 5 min (reverse transcription); 95 °C, 20 s (denaturation) and 95 °C, 10 s + 60 °C, 60 s (45x, amplification).

### Collection of clinical data

In Germany, a reporting requirement for laboratory confirmed infections with IV and RSV is established. Data has been collected at the level of counties and cities. To estimate the association of virus concentration in wastewater and reported cases, the number of infections published by the local public health authorities were used. As IV and RSV infections were not separately reported for the A and B genotypes, the corresponding wastewater data for both IV and RSV (A and B subtypes) was merged. In all catchment areas, we used the cases reported in the county/city in which the WWTP is located. Preliminary analyses resulted in similar course of cases in the counties during the investigation period and a relatively low influence of counties on the epidemiological situation in the great cities. Furthermore, this information about the clinical situation is available on a regular basis allowing evaluations of the epidemiological situation by the authorities and can be easily used without complicated efforts to estimate commuter movements, to optimize the assignment of residents and the time-dependent wastewater flows.

### Data analysis

Normalization of measured virus concentrations in wastewater was done as published^51^. Briefly, flow measurement data and the population in the catchment of the WWTP were provided by the operators of the plants and were used to calculate the viral load per 24 h and capita (dxc). Statistical analyses were performed with GraphPad Prism software (v10.2.3) using standard settings. Differences among groups were considered significant for p-values < 0.05.

## Supplementary Information

Below is the link to the electronic supplementary material.

Supplementary Material 1

## Data Availability

The data underlying this article will be shared on reasonable request to the corresponding author.
