# Supplementary material for: Methodic aspects of influenza and respiratory syncytial virus detection in raw wastewater and presence in treatment plants in southeastern Germany
Source: Sci Rep. 2025 Aug 2;15:28194. doi: 10.1038/s41598-025-13998-x (PMC12317091; doi:10.1038/s41598-025-13998-x)
Supplement: Supplementary file 1 — Supplementary Material 1 [file 41598_2025_13998_MOESM1_ESM.pdf]

Supplementary information

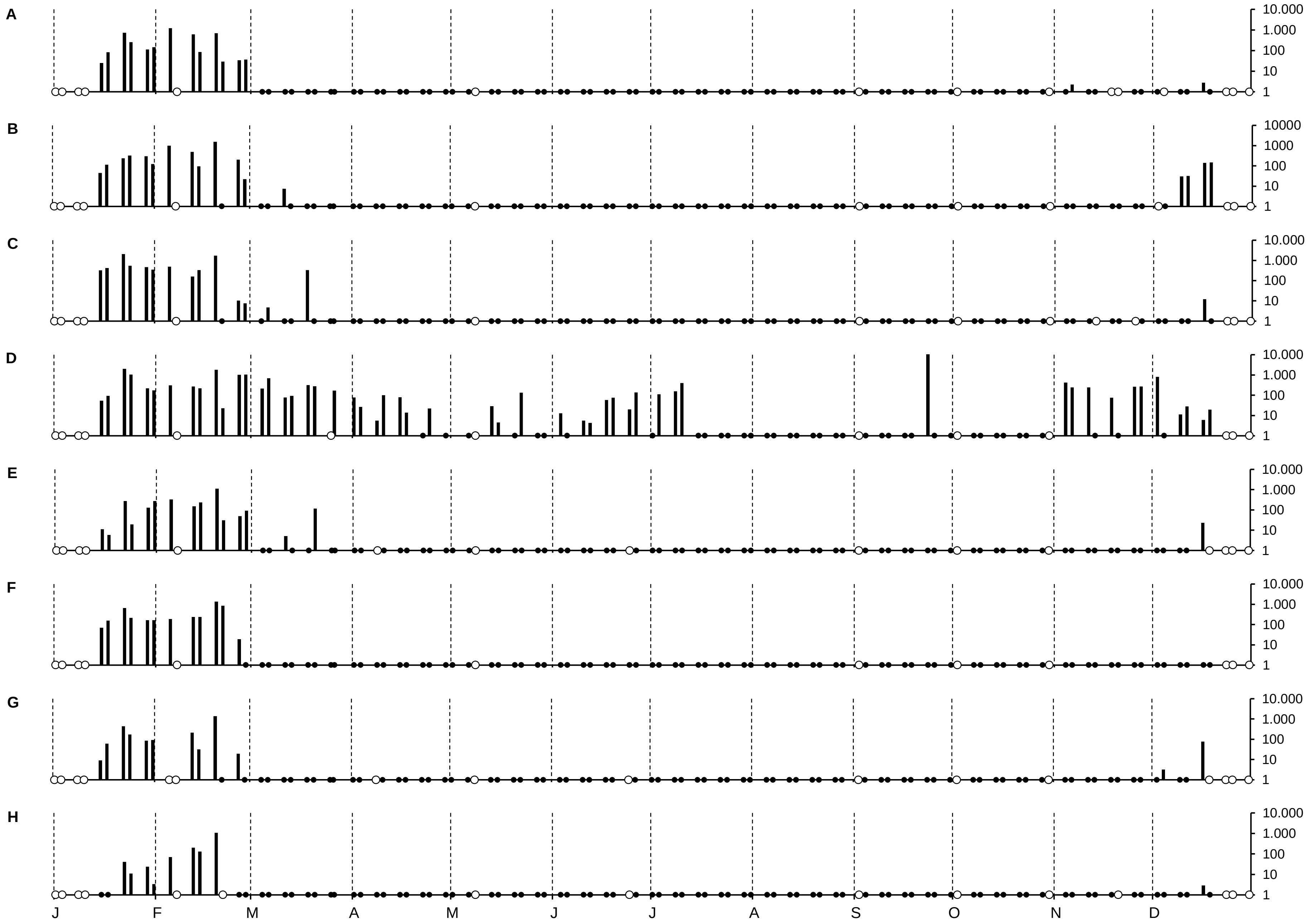

Fig. S1 │ Concentrations of IVA gene copies in wastewater of the WWTPs A-H, 2024. Normalized genome copies per day and capita. Open circles: no sample, filled circle: below detection limit.

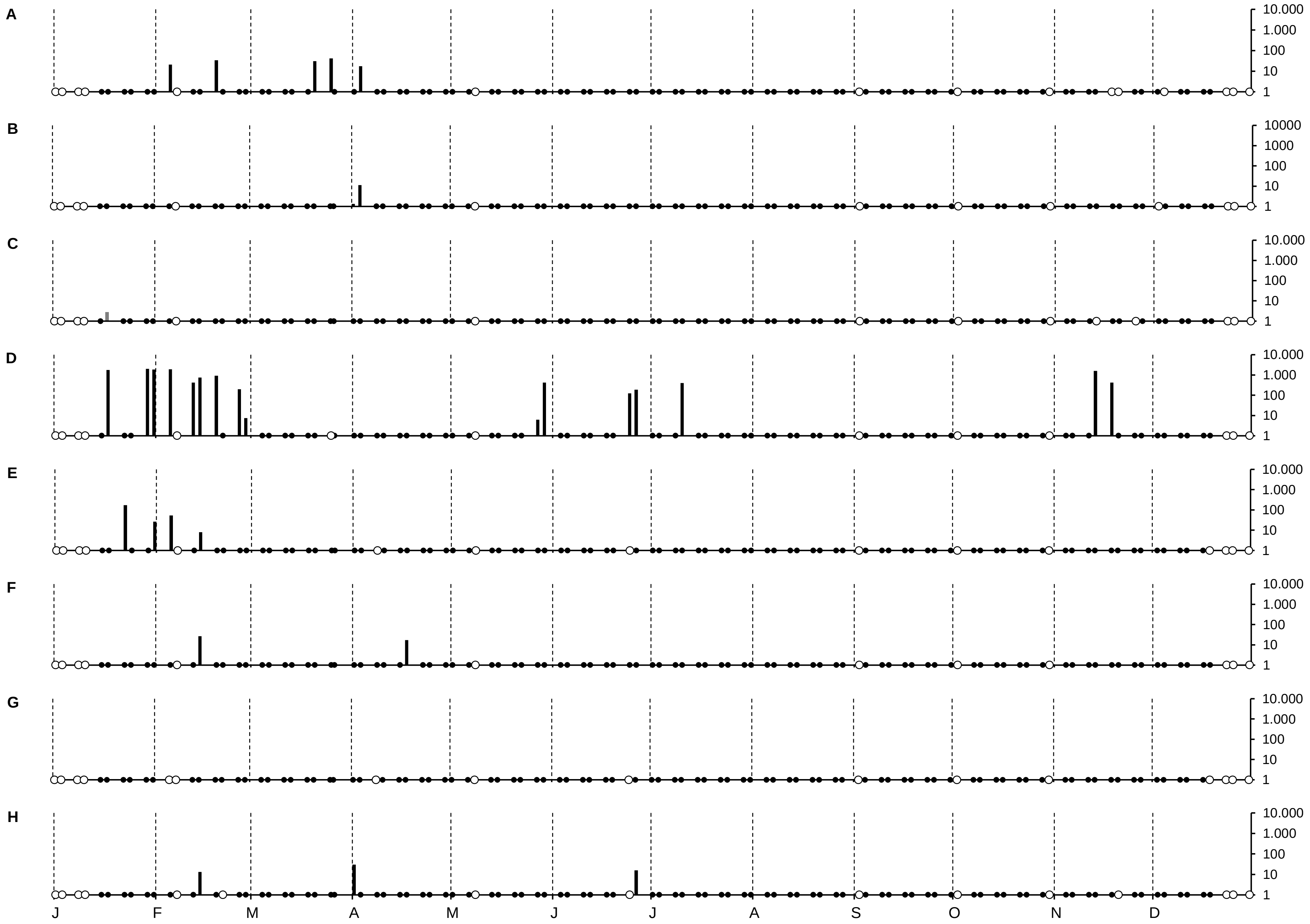

Fig. S2 │ Concentrations of IVB gene copies in wastewater of the WWTPs A-H, 2024. Normalized genome copies per day and capita. Open circles: no sample, filled circle: below detection limit.

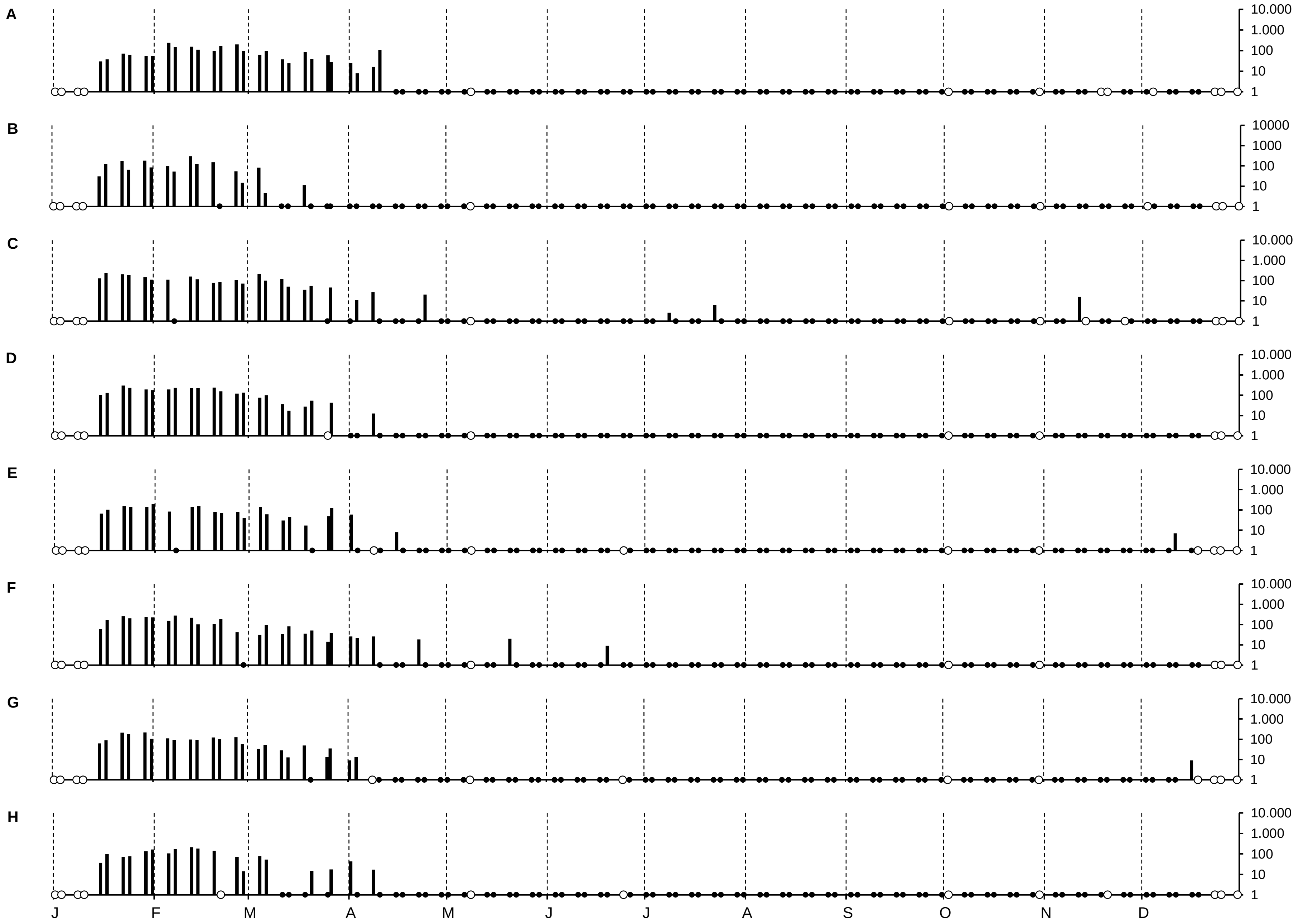

Fig. S3 │ Concentrations of RSV-A gene copies in wastewater of the WWTPs A-H, 2024. Normalized genome copies per day and capita. Open circles: no sample, filled circle: below detection limit.

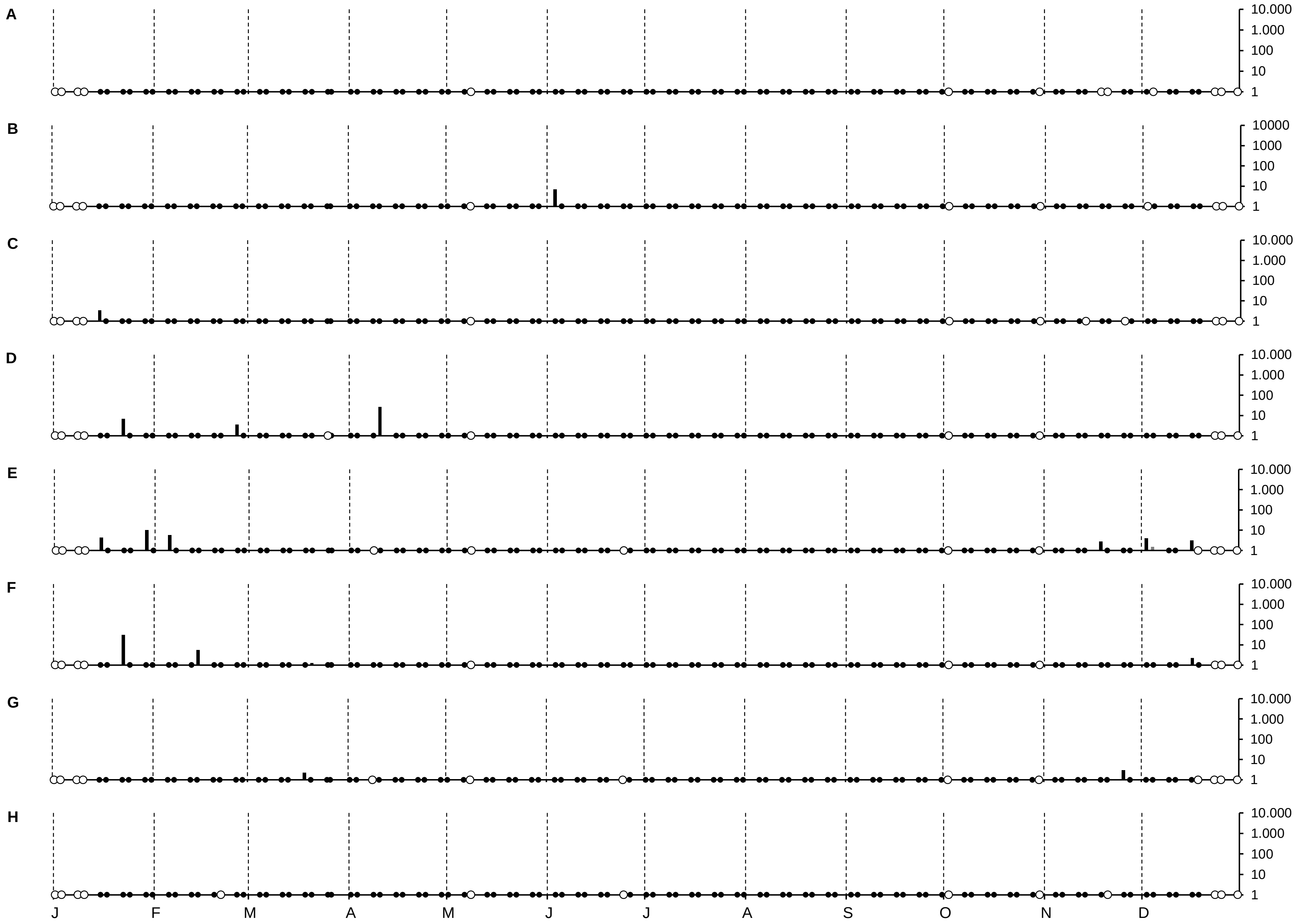

Fig. S4 │ Concentrations of RSV-B gene copies in wastewater of the WWTPs A-H, 2024. Normalized genome copies per day and capita. Open circles: no sample, filled circle: below detection limit.

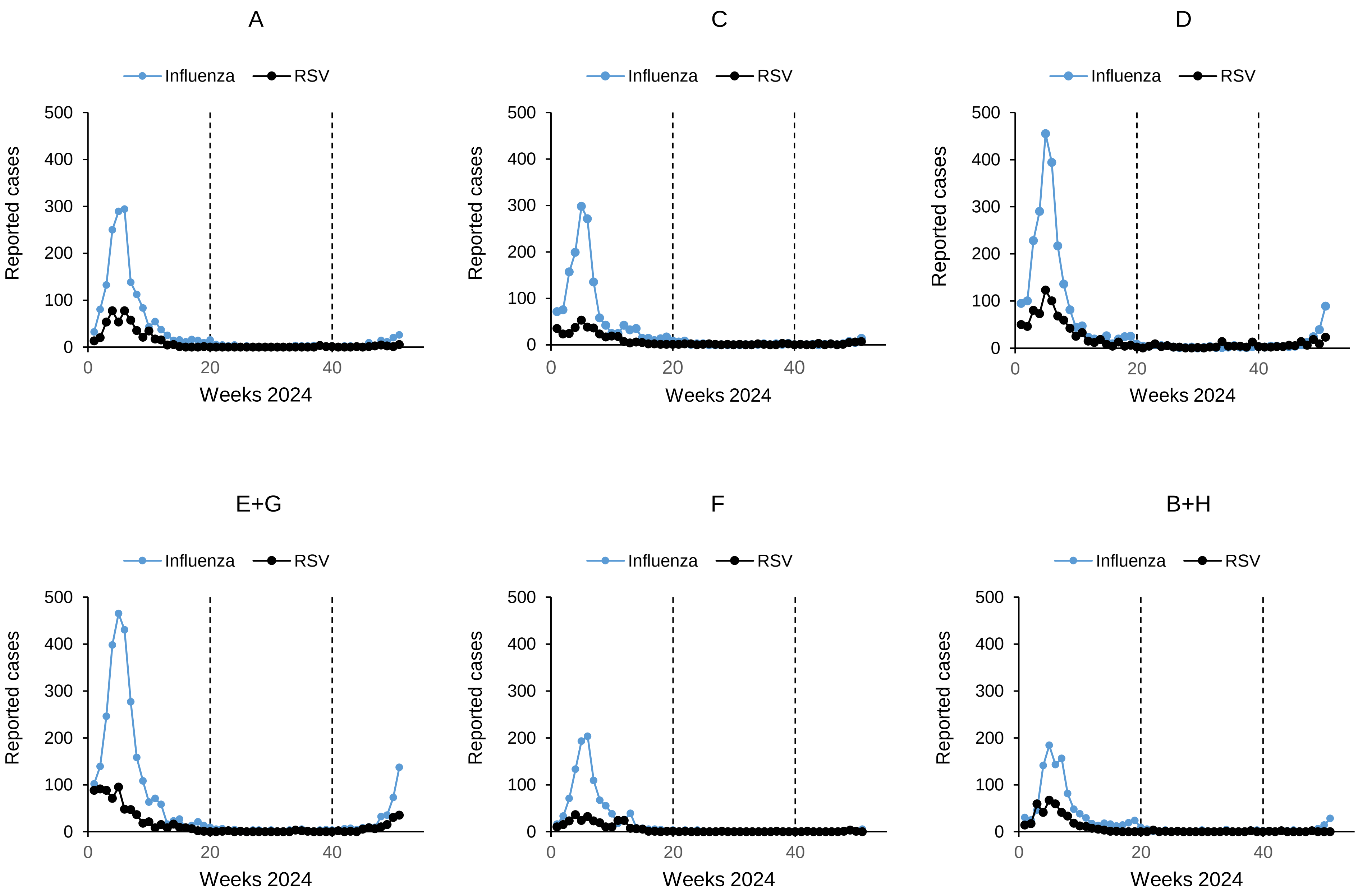

Fig. S5 | Reported cases of laboratory-confirmed influenza and RSV infections in cities and counties in which WWTPs A-H are located (source: https://www.lua.sachsen.de).

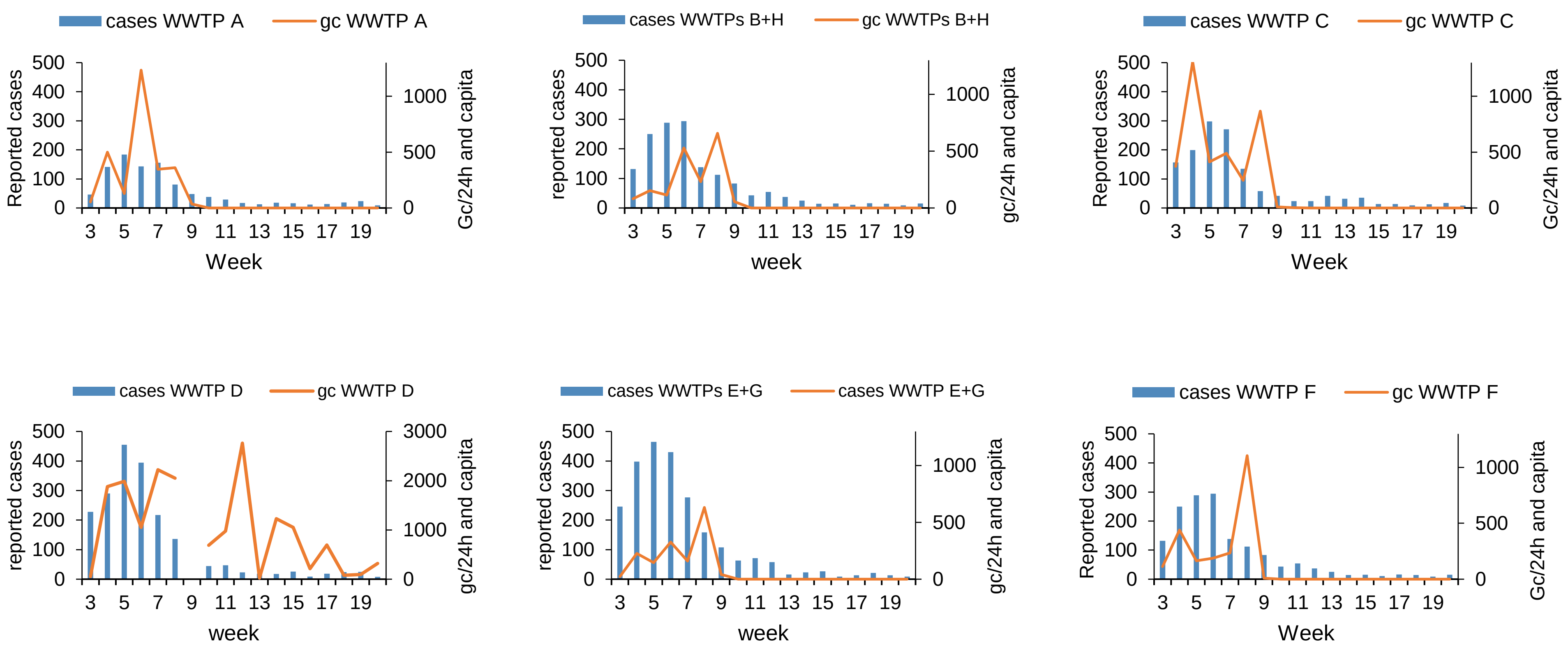

Fig. S6 │ Association of reported cases of laboratory-confirmed influenza infections with measured concentrations of genome copies in wastewater, weeks 3 to 20, 2024.

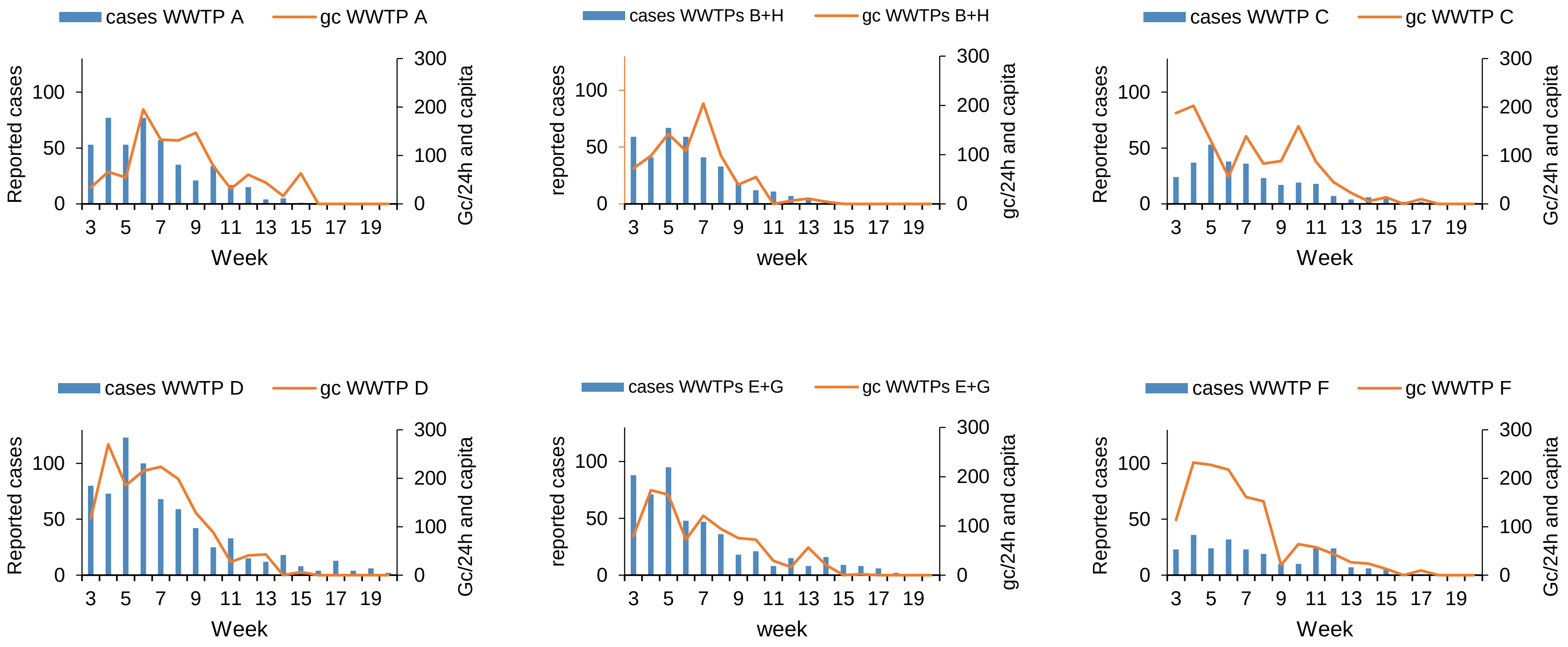

Fig. S7 │ Association of reported cases of laboratory-confirmed RSV infections with measured concentrations of genome copies in wastewater, weeks 3 to 20, 2024.

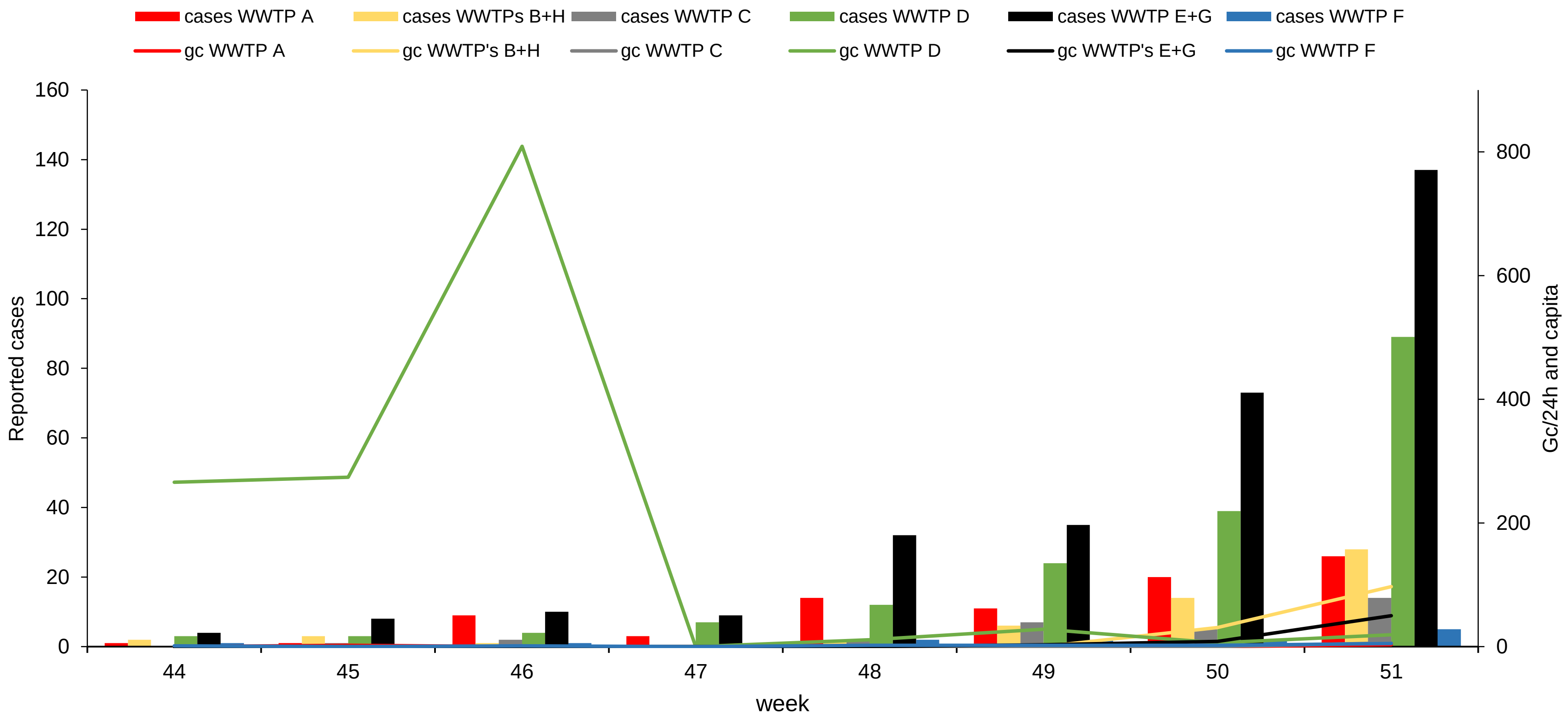

Fig. S8 │ Association between reported cases of influenza infections in the catchments of WWTPs A-H and normalized concentration of viral genome copies (gc) of IV in wastewater during the increase of infections, November-December 2024.

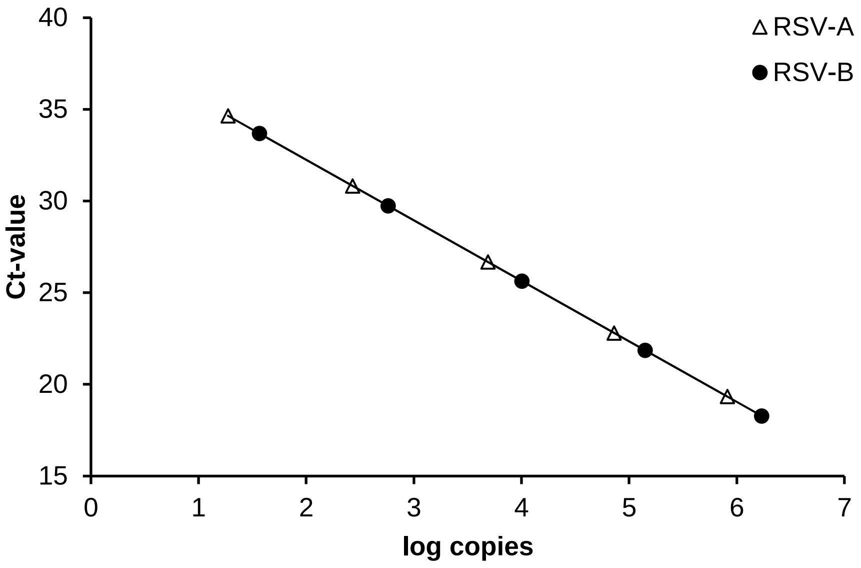

Fig. S9 │ RT-qPCR standard curves for the detection of RSV-A (r^2: 1.000; PCR efficiency: 100.42) and B (r^2: 0.998; PCR efficiency: 101.01), respectively. Arithmetic means and standard deviations of five replicates per dilution.

Table S1 | Statistical significance of differences. A: results of PEG precipitation and other virus concentration procedures (unpaired t-test with Welch's correction). B: normalized number of genome copies in wastewater and reported cases of influenza and RSV in the corresponding catchment areas of WWTPs (p-value and coefficient r of non-parametric Spearman correlation, Fig.5).

A

Virus	PEG-BE	ASP	CEN	WIZ	MagMax	INNU
IV A	0.0029	0.0021	0.0020	0.0364	< 0.0001	0.7992
IV B	0.0026	0.0025	0.0024	0.0055	-	0.0172
RSV A	0.0004	< 0.0001	< 0.0001	0.0419	0.0005	0.0994
RSV B	0.0271	0.0080	0.0151	0.0499	< 0.0001	0.0427

B

WWTP	Influenza		RSV	
	p	r	p	r
A-H	< 0.0001	0.4853	< 0.0001	0.7106
A	0.0004	0.8741	0.0889	0.4931
B+H	0.3956	0.3929	0.1017	0.5883
C	0.0310	0.6930	0.0004	0.8168
D	0.0549	0.3426	0.0102	0.7215
E+G	0.4444	0.3571	0.0023	0.7840
H	0.2675	0.4524	0.0003	0.8452

Table S2 | Limits of detection for the virus monitoring (PEG 8000 precipitation, RNeasy columns, elimination of PCR inhibitors, RealStar detection kits) of IVA/B and RSV-A/B in the investigated eight wastewater treatment plants (10 replicates per dilution).

Virus	R^2	Limit of detection (95%) (copies/45 ml)	Limit of detection (95%) (copies/l)
RSV-A	0.962	213	4.730
RSV-B	0.913	211	4.690
IVA	0.921	400	8.900
IVB	0.920	310	6.900

Table S3 | Primer and probes for the detection of IVA/B in wastewater.

Virus	Primer/probe	Sequence (5'-3')	Reference
Influenza virus A	Forward	GAC CRA TCC TGT CAC CTC TGA C	Lehto *et al*. (2024)
	Reverse	AGG GCA TTY TGG ACA AAK CGT CTA	
	Probe	FAM-TGC AGT CCT CGC TCA CTG GGC ACG-TAMRA	
	Forward	CCM AGG TCG AAA CGT AYG TTC TCT CTA TC	Raya *et al*. (2024)
	Reverse	TGA CAG RAT YGG TCT TGT CTT TAG CCA YTC CA	
	Probe	FAM-ATY TCG GCT TTG AGG GGG CCT G-MGB	
	Forward	CAA GAC CAA TCY TGT CAC CTC TGA C	Boehm *et al*. (2023)
	Reverse	GCA TTY TGG ACA AAV CGT CTA CG	
	Probe	FAM-TGC AGT CCT /Nova/ CGC TCA CTG GGC ACG-BHQ-1	
	Forward	CTT CTA ACC GAG GTC GAA ACG TA	Wolken *et al*. (2022)
	Reverse	GGT GAC AGG ATT GGT CTT GTC TTT A	
	Probe	VIC-TCA GGC CCC CTC AAA GCC GAG-QSY	
Influenza virus B	Forward	TCC TCA AYT CAC TCT TCG AGC G	Boehm *et al*. (2023)
	Reverse	CGG TGC TCT TGA CCA AAT TGG	
	Probe	CIV-550-CCA ATT CGA /BHQ-1/ GCA GCT GAA ACT GCG GTG-C3	
	Forward	AAA TAC GGT GGA TTA AAC AAA AGC AA	Wolken *et al*. (2022)
	Reverse	CCA GCA ATA GCT CCG AAG AAA	
	Probe	FAM-CAC CCA TAT TGG GCA ATT TCC TAT GGC-QSY	
